# Are Familiar Objects More Likely to Be Noticed in an Inattentional Blindness Task?

**DOI:** 10.5334/joc.352

**Published:** 2024-02-22

**Authors:** Yifan Ding, Daniel J. Simons, Connor M. Hults, Rishi Raja

**Affiliations:** 1 Department of Psychology, University of Illinois at Urbana-Champaign, USA

**Keywords:** inattentional blindness, selective attention, familiarity, awareness, noticing, cocktail party effect

## Abstract

People often fail to notice the presence of unexpected objects when their attention is engaged elsewhere. In dichotic listening tasks, for example, people often fail to notice unexpected content in the ignored speech stream even though they occasionally do notice highly familiar stimuli like their own name (the “cocktail party” effect). Some of the first studies of inattentional blindness were designed as a visual analog of such dichotic listening studies, but relatively few inattentional blindness studies have examined how familiarity affects noticing. We conducted four preregistered inattentional blindness experiments (total N = 1700) to examine whether people are more likely to notice a familiar unexpected object than an unfamiliar one. Experiment 1 replicated evidence for greater noticing of upright schematic faces than inverted or scrambled ones. Experiments 2–4 tested whether participants from different pairs of countries would be more likely to notice their own nation’s flag or petrol company logo than those of another country. These experiments repeatedly found little or no evidence that familiarity affects noticing rates for unexpected objects. Frequently encountered and highly familiar stimuli do not appear to overcome inattentional blindness.

Despite our rich and seamless visual experiences, we often fail to notice unexpected objects or events happening in plain sight when we are engaged in an unrelated, attention-demanding task (Mack & Rock, 1998; Neisser & Becklen, 1975; Simons & Chabris, 1999). Undoubtedly, some unexpected objects grab attention more effectively than others. We are unlikely to miss an unexpected object that fills most of the visual field and flashes brightly, for example. Noticing rates also vary as a function of the observer’s attention set, with greater noticing of objects similar to the focus of our attention and less noticing of objects similar to those we are ignoring (Most, Scholl, Clifford & Simons, 2005).

Many studies of inattentional blindness provide evidence that the visual characteristics of the unexpected object can affect noticing (e.g., Most et al., 2001; Wood & Simons, 2017), but few studies have tested whether familiar objects are more likely to be noticed than unfamiliar ones. If they are, that would support a late-selection model in which unattended and unexpected objects are processed richly even when we are not consciously aware of them. In studies of dichotic (selective) listening, people often fail to notice unexpected content in the ignored speech stream, but participants do sometimes notice unexpected but familiar stimuli such as their own names (i.e., the “cocktail party” effect; see Cherry, 1953; Moray, 1959; Wood & Cowan, 1995). Still, many participants did not notice their names or other semantic content in the ignored speech, so it is unclear how fully the ignored content was processed. That is especially true for dichotic listening because participants might well shift attention to the ignored stream on occasion (see Holender, 1986).

Unlike dichotic listening tasks, inattentional blindness tasks typically present an unexpected object that is unrelated to the primary task of attending to and ignoring some display objects. Whereas the unexpected content in dichotic listening is part of the ignore stream, in inattentional blindness tasks, the additional object is entirely unexpected and irrelevant to the primary task, meaning that participants have no task-related reason to anticipate it or to devote attention to it (Mack & Rock, 1998). In that sense, inattentional blindness tasks can provide a stronger test of whether familiarity affects noticing of unattended objects. Several early studies of inattentional blindness explored whether unexpected objects are processed semantically even when they are not noticed (Mack & Rock, 1998). Perhaps the most direct test came from word priming studies (e.g., Mack & Rock, 1998). Participants were asked to look at the fixation mark while judging which arm of a cross flashed briefly in the periphery was longer. On a critical trial, a word unexpectedly appeared at fixation. Participants who did not report seeing the word were more likely to complete a word stem with that word than with another more common completion. However, a more recent registered report study with a much larger sample size and tighter controls on word familiarity found no evidence that unreported words primed stem completion: Only 3 out of 308 participants who missed the unexpected object completed the stem with the less-common prime word (Wood & Simons, 2019).

In another series of studies using the same inattentional blindness task, Mack and Rock (1998) found that participants were more likely to notice their own name unexpectedly presented at fixation than they were a modified version of their name (e.g., “Jack” vs. “Jeck”). Similarly, they reported slightly more noticing of the word “The” than the less common word “Tie.” Both studies tested small numbers of participants, and the difference was not statistically significant.

One other set of studies found evidence for what appears to be a familiarity effect using the same primary task. Participants were more likely to notice an unexpected schematic happy face than a sad, neutral, inverted, or scrambled face (Mack & Rock, 1998). Given that these schematic faces share similar visual features, the difference in noticing may result from people being more familiar with the iconic happy face image than the others. At the time of the study, the happy-face icon was highly familiar and the other schematic faces were less common (see also Wei et al., 2021). A more recent study found greater noticing for all intact faces than for non-faces, partially replicating the earlier result (Redlich, Memmert, & Kreitz, 2022). The lack of a happy-face advantage might reflect the increasing use of neutral and negative face emojis compared to the 1990s.

Overall, there is inconsistent evidence that familiarity contributes to the noticing of unexpected objects. People tended to notice their own names more often, even when presented unexpectedly, but this advantage did not apply to other types of words and was based on small samples. Other word-priming effects did not hold up when tested with larger samples. The benefit for schematic faces over non-faces is, however, consistent with a late-selection model. However, the effect might also reflect an advantage or prioritization specific to faces. Other than the word studies, no studies have examined whether familiar but arbitrary stimuli are more likely to be noticed in inattentional blindness tasks.

Specific words, flags, and logos are arbitrary symbols that become familiar through experience. Unlike faces, they likely do not have any intrinsic features that merit prioritization or preferential processing without such experience. In addition to testing whether we could replicate evidence for greater noticing of certain unexpected faces, we also examined whether established familiarity with arbitrary symbols can affect noticing in an inattentional blindness task.

The four studies in our paper directly measure the effects of familiarity on the noticing of unexpected objects. Experiment 1 replicates studies of inattentional blindness for schematic faces (Mack & Rock, 1998; Redlich et al., 2022) using a larger sample and random assignment to a more complete set of conditions. Experiments 2–4 examine whether familiarity affects noticing by using arbitrary symbols that are more familiar to one set of participants than the other as stimuli.

## General Method and Analysis Procedures

Except as noted, all four experiments used the same task and procedures. Each study was preregistered prior to data collection, and the code, materials, and data are available at https://osf.io/zwcjm/?view_only=f722c5c3d0d64587a6b160cc43cbe9c6. The studies were approved by the Institutional Review Board at the University of Illinois (protocol # 09441) with a waiver of signed consent due to the low-risk, online nature of the experiment. Prior to beginning each experiment, participants viewed an information screen that provided the experimenter and IRB contact information, explained that responses would be anonymous, described how data would be used, and noted that participation was voluntary and compensated.

The studies were conducted online using participants from Prolific. Anyone who had previously participated in one of our studies on Prolific was excluded automatically and could not enroll in the study. We restricted participation to Prolific users who were at least 18 years of age and fluent in English. We did not restrict participants to any region or nationality in Experiment 1 but did for Experiments 2–4.

The experiments adopted a variant of the line-length judgment task originally developed by Mack and Rock (1998) and were coded in Javascript (based on code from Wood & Simons, 2017). An initial instruction screen informed participants that on each trial, they would see a cross appear somewhere on the screen and that they should decide which arm of the cross was longer.

**Figure 1:**
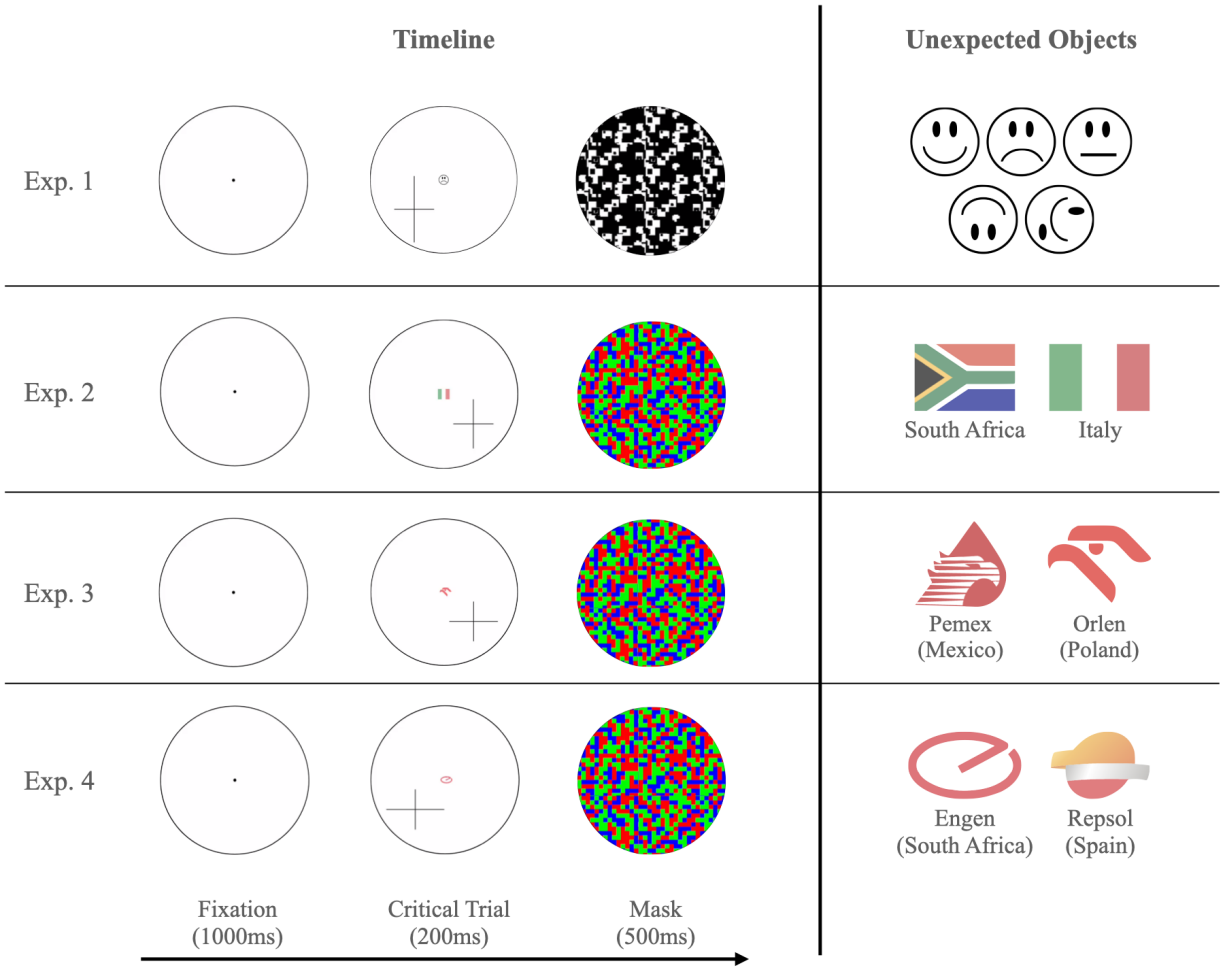
Schematic illustration of a critical trial sequence in Experiments 1–4, along with all possible unexpected objects used in each experiment. The timing of the displays was the same for all four experiments: A fixation dot appeared for 1,000 ms, followed by the cross for 200 ms, and then by a mask. On the critical trial, an unexpected object replaced the fixation dot while the cross was present. The mask in Experiment 1 was black and white because the unexpected objects (face stimuli) were the same colors as well. Experiments 2–4 used a color mask because the unexpected objects were colored (flags in Experiment 2 and petrol company logos in Experiments 3 and 4).

Each trial started with a fixation screen that included a black fixation dot (diameter = 10 pixels) at the center of a black circle (diameter = 500 pixels, thickness = 3 pixels). After 1000 ms, a cross appeared in one of the four randomly selected quadrants of the circle, with the center of the cross positioned 100 pixels vertically and 100 pixels horizontally away from the center of the circle. The arms of the cross were 2 pixels thick, and the length of each arm was chosen randomly from the following four possibilities with the constraint that the two arms differed in length: 135 pixels, 165 pixels, 195 pixels, or 225 pixels. After 200 ms, a pattern mask filled the circle for 500 ms (see Figure 1). Participants were then asked “Which arm of the cross was longer?” with the option to select “The vertical (|) arm” or “The horizontal (—) arm.” Participants then pressed “Continue” to proceed to the next trial.

The first three trials included no unexpected object. On the critical fourth trial, the unexpected object appeared in the center of the circle (where the fixation dot had been) simultaneously with the cross and remained on screen for the same 200 ms duration.

Immediately after reporting which arm of the cross was longer, participants were asked whether they noticed an extra object and then to select it from the possible unexpected objects. They next were asked how frequently they encountered that logo.

Following these questions, participants completed an additional “divided attention” trial that was identical to the critical trial except that the position of the cross was again determined randomly. After reporting which line was longer they answered the same questions as they had for the critical trial. Finally, participants provided demographic information and reported any playback issues. They then were redirected to Prolific to receive payment.

In all four experiments, we aimed to recruit 100 participants for each between-groups condition (which provides sensitivity to reliably detect differences of 10–15% between groups: see Wood and Simons [2017]). After collecting the initial sample, we excluded anyone who reported being under 18 years of age. We did not exclude data for self-reported vision issues or technical problems (we report these preregistered robustness analyses at https://osf.io/zwcjm/?view_only=f722c5c3d0d64587a6b160cc43cbe9c6).

Given a random assignment to conditions, we could not ensure that we would obtain usable data from exactly 100 participants in each condition, so we preregistered stopping rules to guarantee usable data from at least 80 people per condition. Specifically, if we had usable data from fewer than 80 participants in any condition after collecting our target sample size (100 × the number of conditions, k), we identified the condition with the fewest participants and recruited k * (80–N) additional participants. For example, for an experiment with 5 conditions, if after testing 500 people, the condition with the fewest participants had 75 participants, we would schedule an additional 5 * (80–75) = 25 participants, randomly assigning them to conditions in the same manner. We repeated that process until we had usable data from at least 80 participants in all conditions. (Note that we preregistered that we would stop this process if we had not reached that minimum sample size after two weeks, but that time cutoff was not needed for any of the experiments.) With the minimum final sample of 80 participants in a condition, we would have 95% confidence to measure a true noticing rate of 50% within ±10%. (If the true noticing rate were closer to 0 or 100%, the precision would be slightly greater.)

### Analyses

Analyses were conducted in R 4.3.1 (R Core Team, 2023) using tidyverse 2.0.0 (Wickham, 2023). The manuscript was written in RMarkdown 2.24 (Allaire et al., 2023) using papaja 0.1.1 (Aust & Barth, 2022), ggplot2 3.4.3 (Wickham et al., 2023), ggimage 0.3.3 (Yu, 2023), ggpattern 1.0.1 (FC, Davis, & ggplot2 authors, 2022), png 0.1.8 (Urbanek, 2022), reshape2 1.4.4 (Wickham, 2020), knitr 1.43 (Xie, 2023), and kableExtra 1.3.4 (Zhu, 2021).

The primary analysis scripts were written prior to data collection (see preregistration). We report all data exclusions, measures, and manipulations here and in the preregistration (Simmons, Nelson, & Simonsohn, 2011). Following our practice in previous studies of inattentional blindness (see Wood & Simons, 2019), we did not conduct frequentist statistical analyses. In most cases, the pattern of results for inattentional blindness studies is clear from visual inspection.

**Figure 2:**
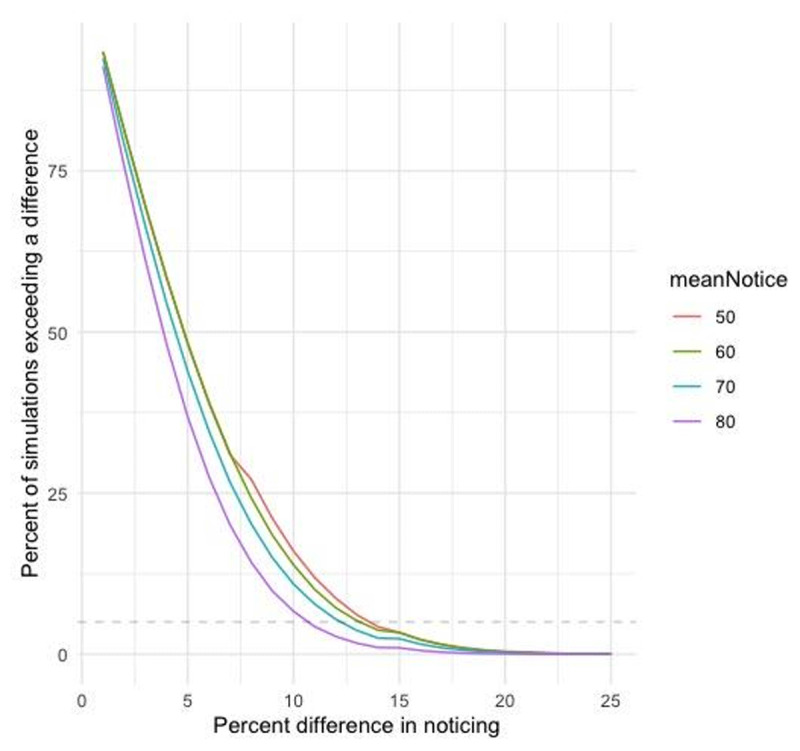
Simulation illustrating the likelihood of differences in noticing rates of various magnitudes as a function of the mean noticing rate across the two groups (with no actual difference in noticing between the two groups). The horizontal dashed line is at 5%, so differences in noticing falling below that line would be expected less than 5% of the time if there actually were no differences between groups. The graph shows average noticing rates above 50%, but the pattern would be the same for averages less than 50% based on how far they fall from 50% (e.g., the results for an average of 40% would be the same as for an average of 60%).

Given that percentage differences in noticing are already meaningful effect size measures, we did not convert them to standardized effect sizes. To estimate how frequently we would expect differences in noticing rates of a given magnitude if there were no differences in reality, we recently (Ding, Hults, Raja, Simons, 2023) simulated 100,000 inattentional blindness studies, each with 2 conditions with a true noticing rate of 50% (100 participants in each). Across those simulations, a difference of 10% occurred approximately 16.0% of the time and a difference of 15% occurred approximately 3.3% of the time. With noticing rates closer to 0% or 100%, differences that large were less common (see Figure 2, reproduced from Ding et al., 2023).

Based on these simulations, Our preregistration specified that we would treat conditions differing in noticing by less than 10% as roughly comparable and conditions differing by 10–15% as a small effect. For each experiment, we preregistered predictions for how familiarity might affect noticing, and we compared the observed results to those patterns.

## Experiment 1

**Figure 3:**
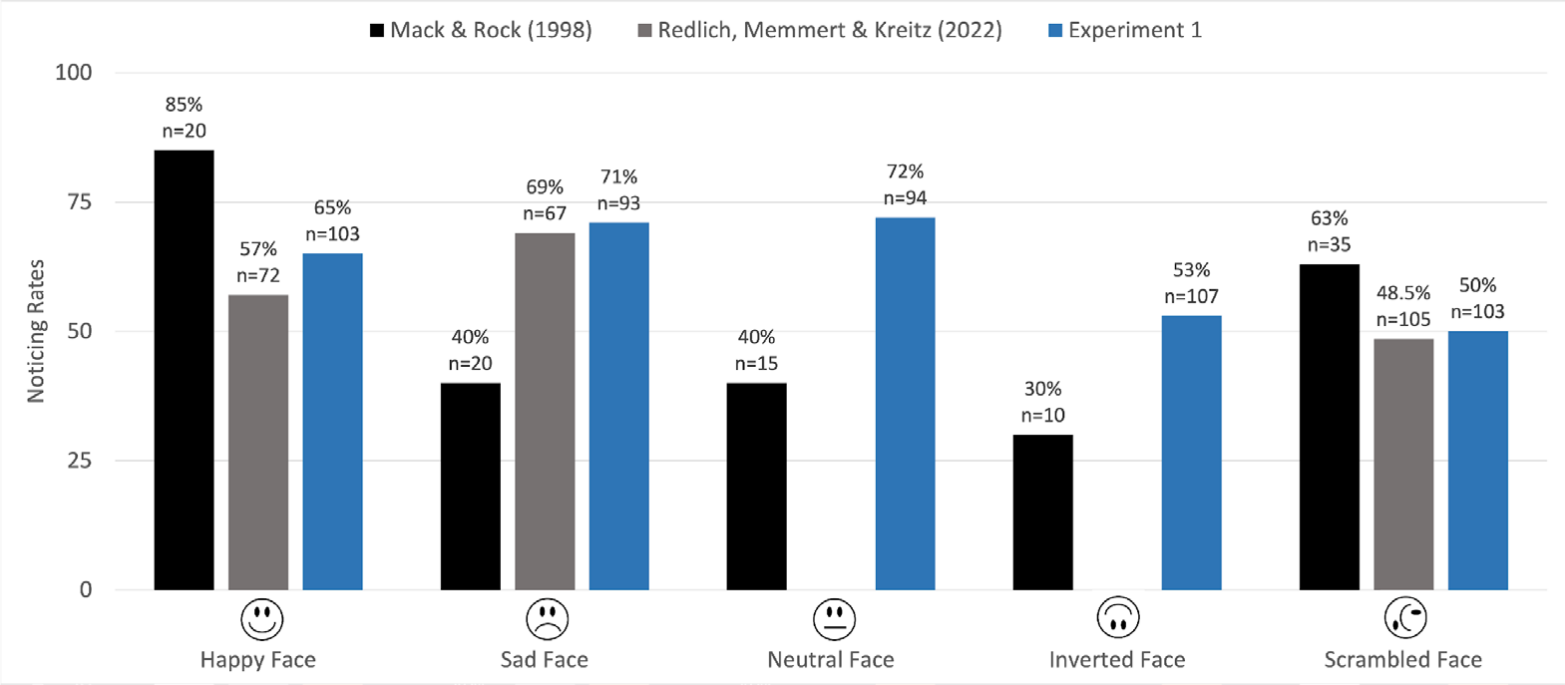
Pattern of noticing for Experiment 1 along with earlier results with schematic faces from studies by Mack & Rock (1998) and Redlich, Memmert, & Kreitz (2022).

This experiment replicated studies by Mack and Rock (1998) and Redlich, Memmert, and Kreitz (2022) that examined the effect of facial expression on inattentional blindness. Mack and Rock (1998) observed higher noticing rates for an unexpected smiling face than for sad, neutral, inverted, or scrambled faces (see Figure 3; note that they collected data for these conditions across several experiments). Redlich, Memmert, and Kreitz (2022, experiment 1) used a similar design, except that the cross appeared at fixation and the unexpected object appeared in one of the four quadrants of the display. They found slightly higher noticing rates for sad faces than happy faces, with both of those schematic faces noticed more than scrambled faces.

### Predictions

Given that people likely are more familiar with happy, sad, and neutral schematic faces than with inverted or scrambled faces, if familiarity increases noticing, the intact, upright faces should be noticed more than the scrambled or inverted ones. Note that the noticing rates for happy, sad, and neutral faces might differ if they are not equally familiar to participants. The inverted face also might be noticed more than the scrambled face if it is more familiar.

### Method

Following our general procedures, we aimed to recruit 500 age-eligible participants and randomly assigned each to one of five possible unexpected objects: happy, sad, neutral, inverted, or scrambled face (see Figure 1). Following our stopping rules, our final sample included usable data from 503 participants. Each participant received $0.30 for completing the study.

Each unexpected object was 40 pixels in width and 40 pixels in height. Given that the unexpected stimuli were black and white, the 500 ms pattern mask was composed of scrambled black and white pixels (see Figure 1).

### Results

Our primary analysis included data from all 500 participants (all aged 18 or older) who completed the study. Figure 3 shows the number of participants and noticing rate for each condition.

Noticing rates for happy, sad, and neutral faces were roughly comparable: All three faces were noticed by approximately two-thirds of participants. In contrast, inverted and scrambled faces were noticed by approximately half of the participants.

### Discussion

This pattern of results replicates evidence that schematic faces are more likely to be noticed than inverted or scrambled faces (Mack & Rock, 1998; Redlich et al., 2022). Like Redlich et al. (2022), we did not replicate evidence that happy faces are noticed more than sad or neutral faces (Mack & Rock, 1998); noticing was roughly comparable for happy, sad, and neutral expressions.

One possible explanation for the failure to observe a happy-face advantage both in our study and that by Redlich et al. (2022) is that the original finding of a happy-face advantage was a false positive result. A plausible alternative, however, is a cohort effect. The original study was conducted in the 1990s, well before face emojis were common. At that time, only the canonical smiley face was commonly used, so Mack and Rock’s participants might well have been more familiar with it than with other types of faces. Unfortunately, there is no easy way to examine whether the differential familiarity of face emojis in the subject populations tested in the 1990s explains the failure to replicate the happy-face advantage. However, we can examine whether familiarity decreases inattentional blindness using other types of stimuli and by testing populations that vary in their familiarity with them. The remaining studies in this paper test whether familiarity affects noticing by fully crossing the type of stimulus with the demographics of the sample.

## Experiment 2

Experiments 2–4 each tested participants from a pair of regions with stimuli that were or were not familiar in each region (or at least differed in familiarity between them). By fully crossing the stimulus and region, we ensured that differences in noticing result from differences in familiarity rather than differences in the features of the stimuli themselves. Experiment 2 tested participants from Italy and South Africa using those country’s national flags. Experiments 3 and 4 used pairs of corporate logos that were familiar in one country but not another.

### Predictions

If familiarity affects noticing of the unexpected object, noticing rates of a flag should be higher for participants in the country it belongs to (e.g., higher noticing rates of the Italian flag for Italian than South African participants; higher noticing rates of the South African flag for South African than Italian participants). The overall noticing rates might differ for the two flags based on how noticeable their visual features are, but that sort of overall stimulus difference is not of interest given that the flags are not otherwise equated for these features.

### Method

The Italian and South African flags both are red, green, and white, but they should differ in familiarity for participants from those countries (the flags were 50 pixels wide by 40 pixels high). Given that both flags are colored, we used a color pattern mask consisting of scrambled red, green, yellow, black, and white pixels (see Figure 1).

We posted two experiments on Prolific simultaneously, one recruiting Italian participants and one recruiting South African participants. All participants received $0.56 for completing the study. Participants in each country were randomly assigned to see either the Italian flag or the South African flag (and saw the same flag on both the critical and divided attention trials). We aimed to collect 200 age-eligible participants from each country and applied the stopping rules described in the general procedures section. In total, we collected usable data from 402 participants (200 Italian and 202 South African) after excluding data from one participant who reported being younger than 18. Given that participants should have been at least 18 years old based on the experiment requirements in Prolific, it is unclear why this younger participant was in the pool. It is possible that the participant actually was 18 or older but selected the wrong option in response to our age question.

### Results

**Figure 4:**
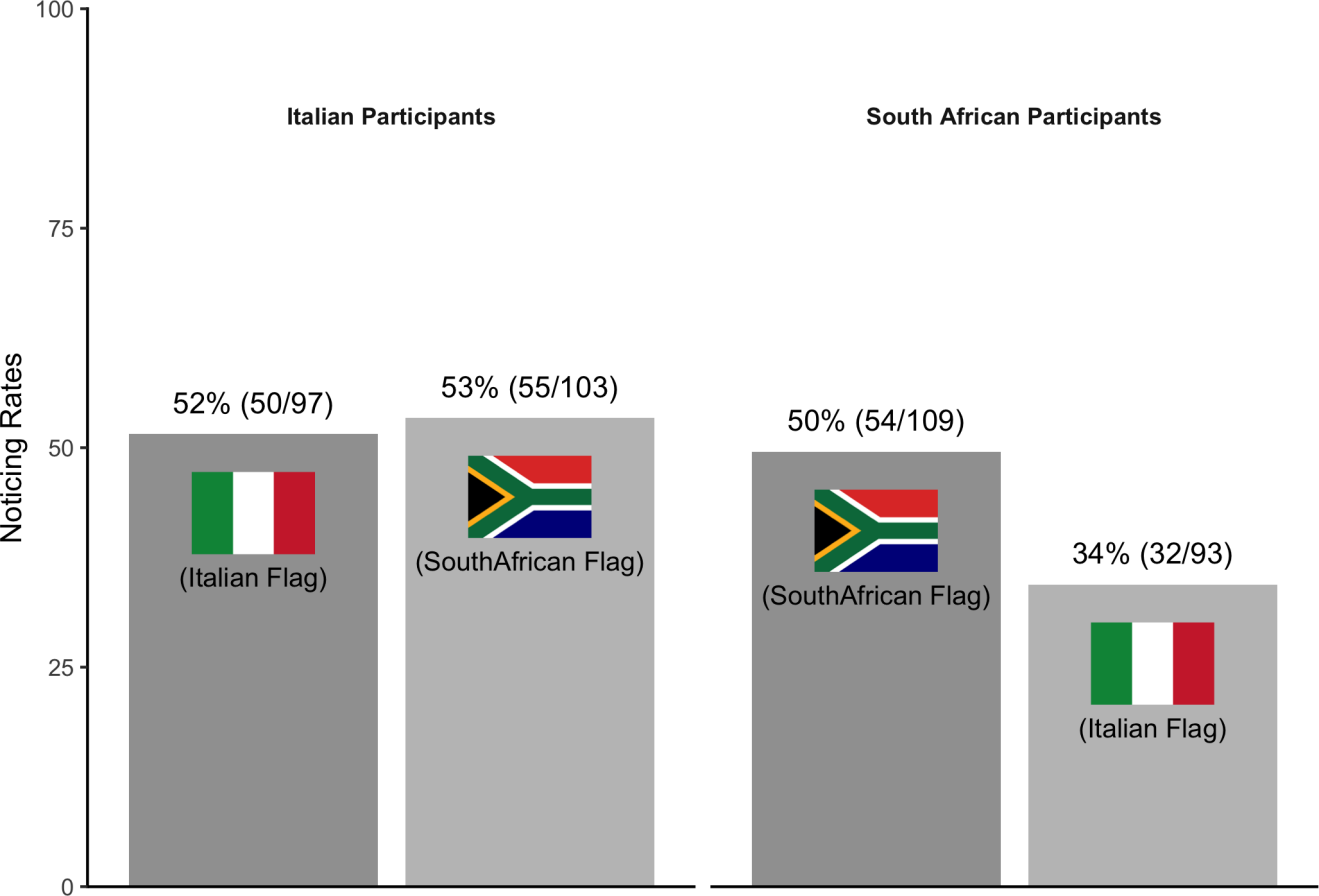
Pattern of noticing for Experiment 2.

Italian participants noticed both the Italian flag and the South African flag at comparable rates, suggesting no effect of familiarity on noticing (Figure 4). South African participants, however, were more likely to notice the South African flag than the Italian flag. Thus, the study provides inconsistent evidence for an effect of familiarity on noticing.

As part of their questioning after the critical and divided attention trials, participants were asked to select the flag they had seen, but neither flag was labeled. In the divided attention trial, most participants saw something. If they spontaneously named the other country’s flag when describing what they saw, that implies they were familiar with it and knew what it was. For example, if an Italian participant was shown the South African flag and on the divided attention trial, they specifically stated that the “unexpected” object was the South African flag, that shows they knew what the flag was prior to the study, so it was not unfamiliar to them.

**Figure 5:**
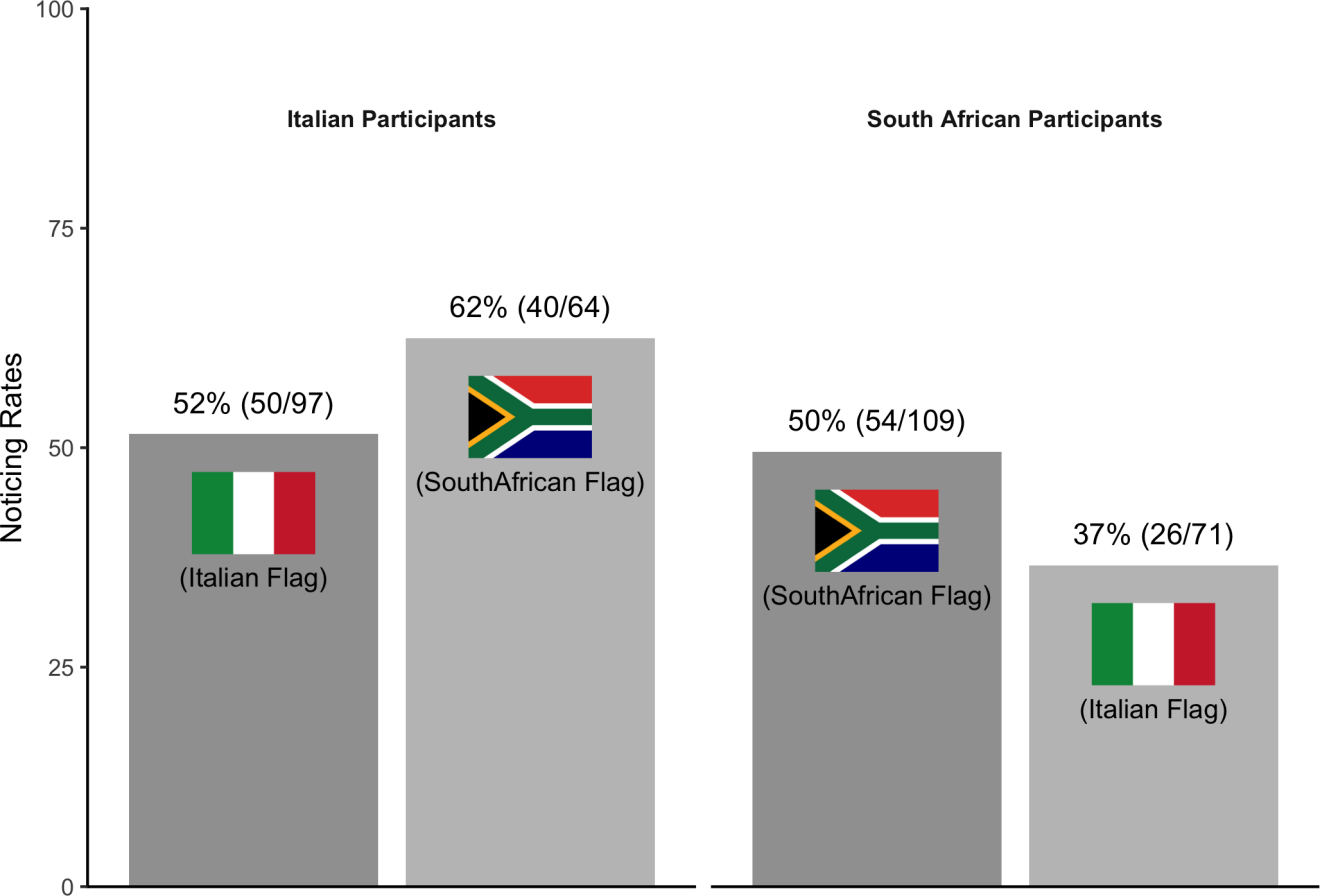
Pattern of noticing for Experiment 2 – exploratory analysis.

In an exploratory analysis, we excluded data from Italian participants who, on the divided attention trial, correctly named what they saw as the South African flag (n = 39) and South African participants who correctly and spontaneously labeled what they saw as the Italian flag (n = 22). Combining across countries, familiar flags now were no more likely to be noticed (50%; 104/206) than unfamiliar flags (49%; 66/135; see Figure 5).

## Experiment 3

To eliminate the possibility that national flags did not provide a strong test of familiarity effects due to greater than anticipated familiarity with flags from other countries, Experiment 3 used a pair of company logos that occur exclusively in one country and not another. In many countries, nearly all petrol stations have a single brand and logo. We tested participants from Mexico, who would be familiar with the Pemex logo, and Poland, where they would be familiar with the Orlen logo.

### Predictions

If familiarity affects noticing of the unexpected object, noticing rates should be higher for participants presented with the logo of their own national petrol company (e.g., higher noticing of the Pemex logo for Mexican participants; higher noticing of the Orlen logo for Polish participants). Again, it is possible that one logo will be noticed more than the other, but for the present purposes, we are not interested in differences due to the noticeability of the stimuli themselves as opposed to those due to differences in familiarity across participants.

### Method

We ran two experiments on Prolific simultaneously, one with Mexican participants and one with Polish participants. Each participant received $0.50 for completing the study. Participants in each country were randomly assigned to see either the Pemex logo (Mexico) or the Orlen logo (Poland) and saw the same logo on both the critical and divided attention trials. Both logos are red with similar bird-like contours and were shown at 65% opacity with a width of 50 pixels and a height of 40 pixels (see Figure 1). We used the same colored mask as in Experiment 2.

We aimed to collect 200 age-eligible participants from each country and applied the stopping rules described in the general method section. In total, we collected usable data from 399 participants, with 200 Mexican and 199 Polish participants.

### Results

For the primary analysis, we included data from all 399 participants who completed the study (none reported being younger than 18).

**Figure 6:**
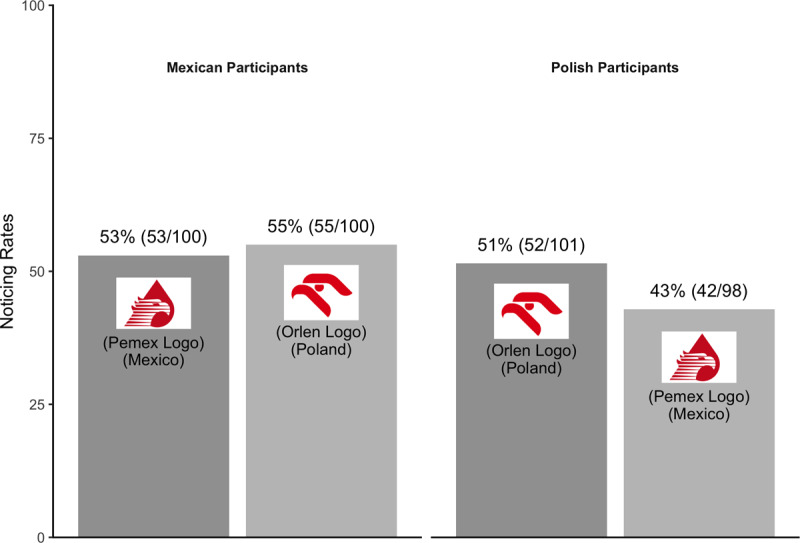
Pattern of noticing for Experiment 3.

Mexican participants noticed both the Mexican Pemex logo and the Polish Orlen logo at comparable rates, suggesting no effect of familiarity on noticing (Figure 6). Polish participants were only slightly more likely to notice the familiar Orlen logo than the unfamiliar Pemex logo. Overall, the study provides little evidence for an effect of familiarity on noticing.

For a secondary, preregistered analysis, we excluded data from participants who reported some familiarity with the company logo from the other country. Specifically, we excluded Mexican participants who were shown the Polish Orlen logo, reported noticing something on the divided attentional trial, and reported anything other than “rarely or never” for how frequently they encountered the Polish Orlen logo (n = 14). And, we excluded data from Polish participants who were familiar with the Mexican Pemex logo using the same criteria (n = 7).

**Figure 7:**
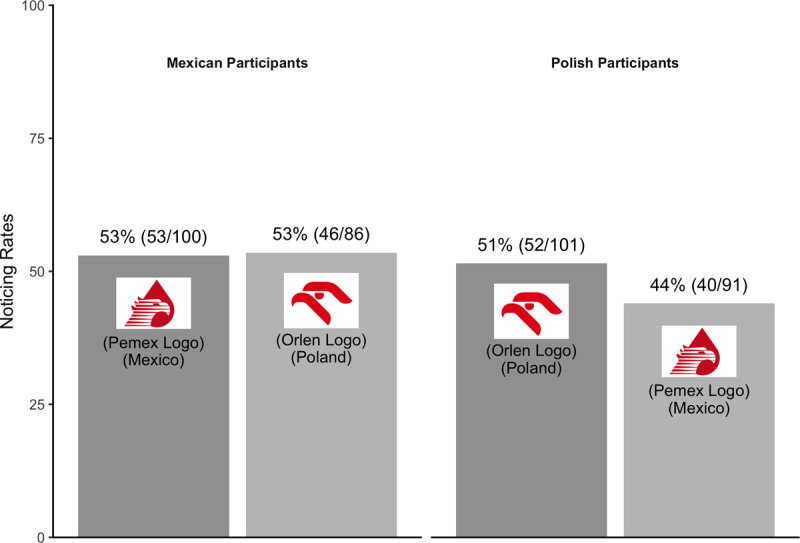
Pattern of noticing for Experiment 3 – secondary analysis.

Excluding these participants did not change the pattern of noticing from the primary analysis, suggesting no overall effect of familiarity. Combining across countries, familiar logos were no more likely to be noticed (52%; 105/201) than unfamiliar logos (49%; 86/177; see Figure 7).

**Figure 8:**
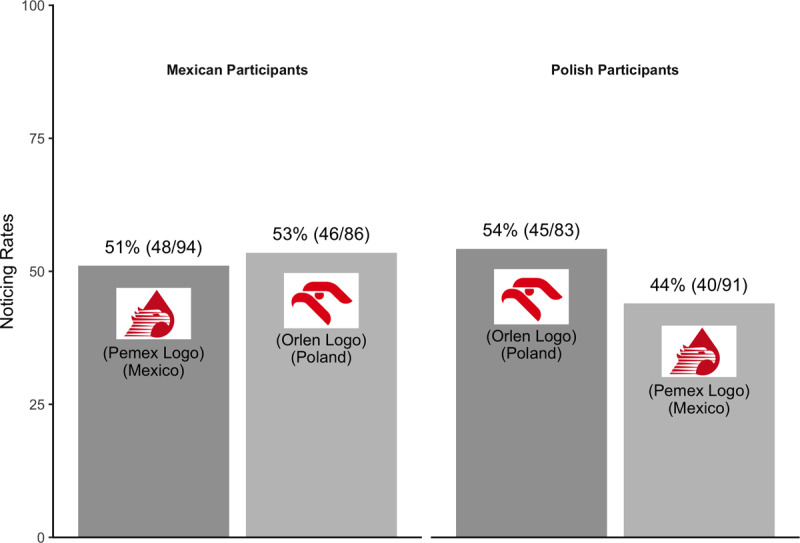
Pattern of noticing for Experiment 3 – exploratory analysis.

Just as there were participants familiar with another country’s logo, some might be unfamiliar with their own country’s logo. For a further, exploratory analysis, we excluded participants who were shown their own country’s logo, noticed the critical object on the divided attentional trial, and reported seeing their own country’s logo “rarely or never” (Mexican participants: n = 6; Polish participants: n = 18). After excluding data from these participants as well, there still was no evidence of an effect of familiarity on noticing (see Figure 8). Combining across countries, familiar logos were not substantially more likely to be noticed (53%; 93/177) than unfamiliar logos (49%; 86/177).

## Experiment 4

Experiment 4 attempted to replicate the results of Experiment 3 using participants from South Africa, where the national petrol brand is Engen, and Spain, where the national petrol brand is Repsol.

### Predictions

As in Experiment 3, if familiarity affects noticing of the unexpected object, noticing rates should be higher for participants presented with the logo of their own nation’s company (e.g., higher noticing rates for South African participants with the South African Engen logo than the Spanish Repsol logo).

### Method

We ran two experiments on Prolific simultaneously, one with South African participants and one with Spanish participants. Each participant received $0.50 for completing the study. Participants in each country were randomly assigned to see either the South African Engen logo or the Spanish Repsol logo (and saw the same logo on both the critical and divided attention trials). The Engen and Repsol logos were shown at 65% opacity with a width of 50 pixels and a height of 40 pixels (see Figure 1). We used the same colored mask as in Experiment 2.

We aimed to collect 200 age-eligible participants from each country and applied the stopping rules described in the general method section. In total, we collected usable data from 400 participants, with 203 South African and 197 Spanish participants.

### Results

**Figure 9:**
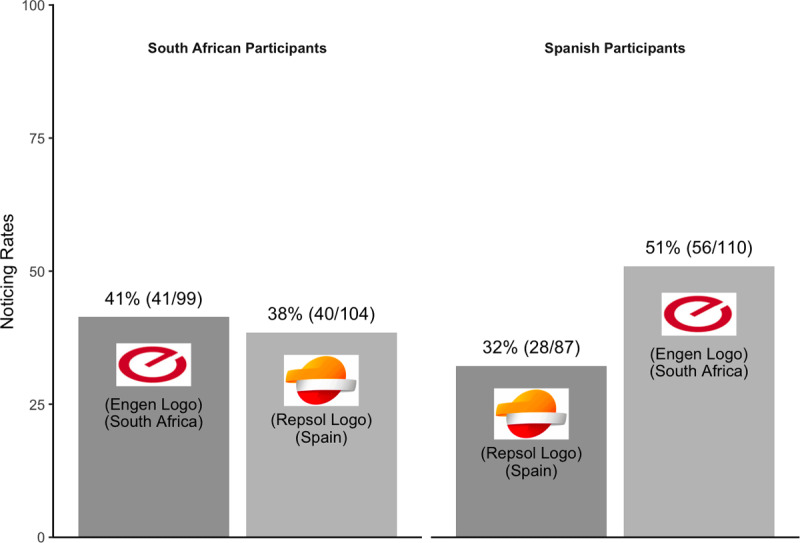
Pattern of noticing for Experiment 4.

All participants again reported being older than 18, so all 400 participants were included in the primary analysis. South African participants noticed both the South African Engen logo and the Spanish Repsol logo at comparable rates, suggesting no effect of familiarity on noticing (Figure 9). Spanish participants, however, were more likely to notice the unfamiliar South African Engen logo than the familiar Spanish Repsol logo. This pattern is not consistent with greater noticing of familiar stimuli.

**Figure 10:**
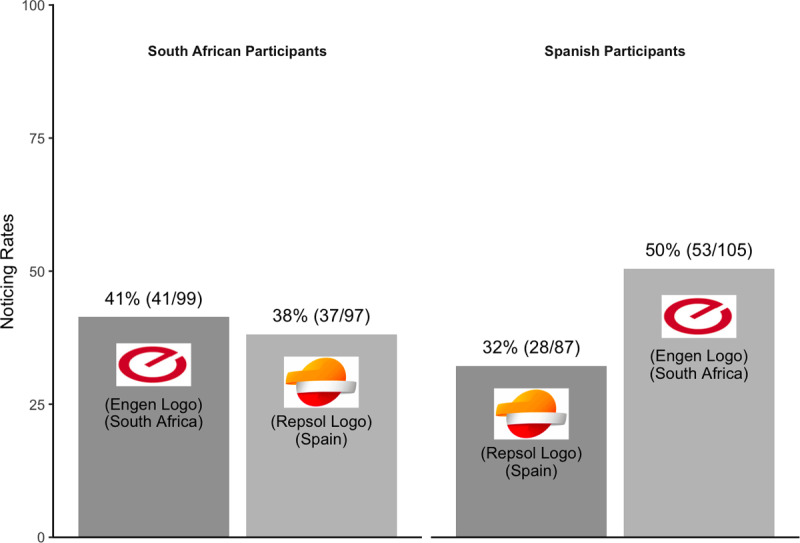
Pattern of noticing for Experiment 4 – a secondary analysis.

For a secondary, preregistered analysis, we again excluded data from South African participants who were shown the South African Engen logo, reported noticing the critical object on the divided attentional trial, and reported anything other than “rarely or never” for how frequently they encountered the South African Engen logo (n = 7). And, we excluded data from Spanish participants who were familiar with the Spanish Repsol logo using the same criteria (n = 5). Excluding these data did not affect the pattern of noticing from the primary analysis. Combining across countries, familiar logos were no more likely to be noticed (37%; 69/186) than unfamiliar logos (45%; 90/202). Spanish participants were still more likely to notice the unfamiliar Engen logo than the familiar Repsol one (Figure 10).

**Figure 11:**
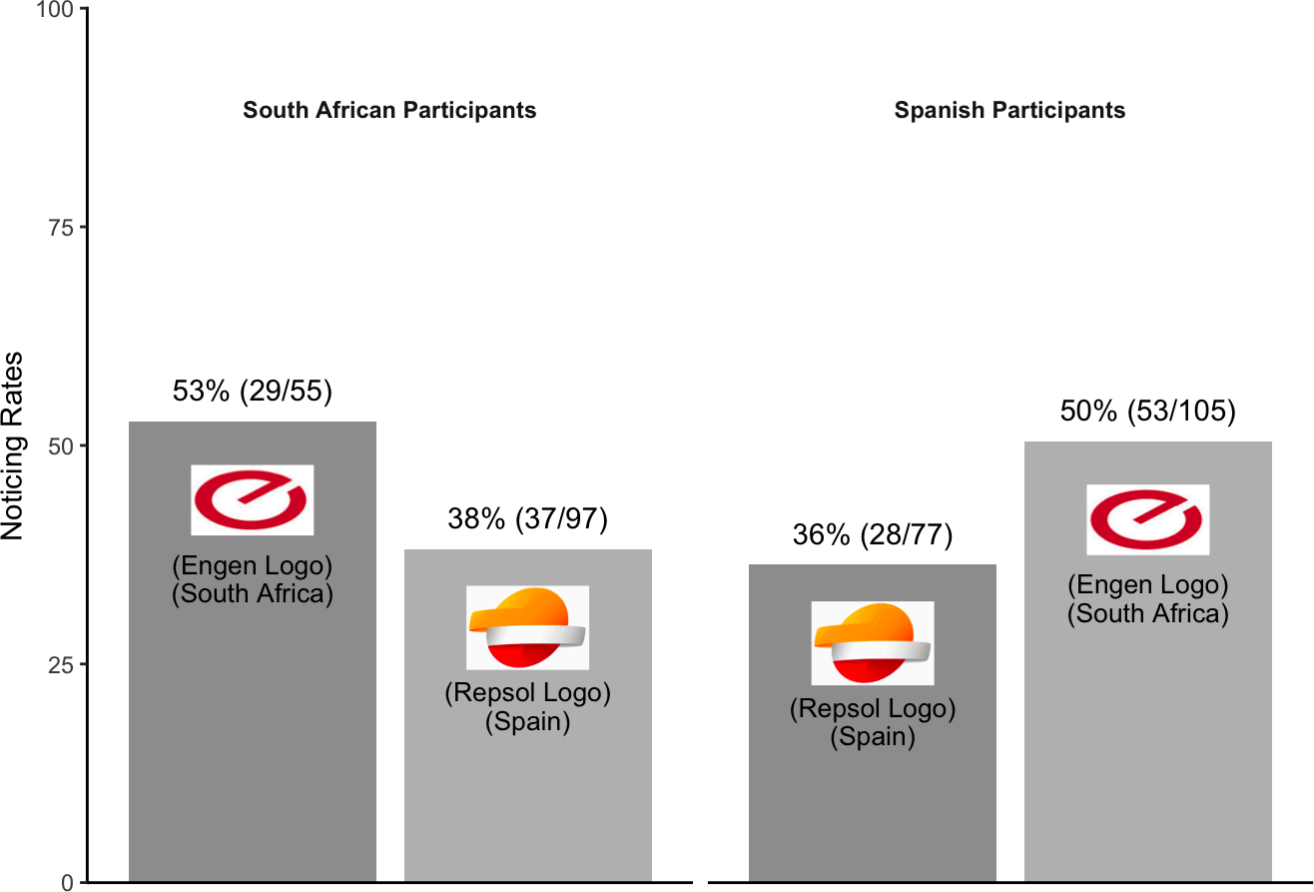
Pattern of noticing for Experiment 4 – an exploratory analysis.

As in Experiment 3, for an exploratory analysis, we also excluded data from participants who reported seeing their own country’s logo rarely or never (South African participants: n = 44; Spanish participants: n = 10). Doing so eliminated the small advantage for unfamiliar logos (Figure 11). Combined across countries, familiar logos were no more likely to be noticed (43%; 57/132) than unfamiliar logos (45%; 90/202).

## General Discussion

Across four experiments using a static cross-judgment inattentional blindness task, we found no consistent evidence that familiarity with an unexpected object affects the likelihood of noticing it. Experiment 1 replicated Redlich, Memmert, and Kreitz (2022) and found that schematic faces were more likely to be noticed than non-schematic faces. Like Redlich et al., we did not replicate the original Mack and Rock (1998) finding of greater noticing of schematic happy faces.

One explanation for greater noticing of standard schematic faces than inverted or scrambled ones is that familiarity decreases inattentional blindness. But that effect of familiarity might be specific to faces or other naturalistic stimuli. To examine that possibility, we also tested whether familiarity affects noticing of more arbitrary symbols in Studies 2–4.

Experiment 2 tested participants from Italy and South Africa and used the national flags of those countries as stimuli, fully crossing the unexpected object and the participant country. Overall, participants were no more likely to notice their own country’s flag than that of another country. One limitation of using national flags is that people vary in their knowledge of the flags of other countries. Experiments 3 and 4 replicated the cross-over design of Experiment 2 using pairs of national petrol logos that should be familiar to people within one country but not another. We again found no effect of familiarity: Participants were no more likely to notice the logo common in their own country than the logo common in another country.

Collectively, results from Experiments 2–4 undermine support for a visual analog of the cocktail party effect: When attention is focused on a primary task, the familiarity of an unexpected flag or logo does not appear to increase whether or not people notice it. In contrast, for schematic faces, we did find an effect of familiarity, suggesting that faces differ from more arbitrary symbols with learned meanings. The idea that faces might be special is consistent with evidence that faces are better detected than common objects or inverted faces (Devue, Laloyaux, Feyers, Theeuwes, & Brédart, 2009). But the effect of familiarity for faces in our studies might also reflect a difference in the effects of familiarity for objects with inherent meaning and those with an arbitrary, learned mapping between visual features and meaning. Future studies could further examine whether upright faces truly are special in their greater ability to elude inattentional blindness.

In sum, we found no consistent effect of familiarity on inattentional blindness for flags or logos, but we did observe greater noticing of upright, schematic faces than inverted or scrambled ones. This suggests that familiarity with arbitrary objects might not lead to awareness in the absence of attention, but familiarity with natural and potentially important stimuli such as faces might lead to greater processing without deliberate attention, consistent with a late-selection model of attention (Deutsch & Deutsch, 1963). Future studies should examine whether the pattern observed for schematic faces occurs for any other natural objects or only for faces.

### Constraints and Limitations

We expect our pattern of results would generalize to inattentional blindness studies with similar designs. It is unclear, though, whether we would observe a familiarity effect in a dynamic inattentional blindness task, in which the unexpected object persists for an extended period of time while also moving across the display. Given that inattentional blindness studies have observed similar results online and in the lab, we have no reason to expect the results would differ with in-person testing. We would also expect our results to generalize to other pairs of countries and logos, given that we observed comparable results for flags and logos from three pairs of countries.

## Data Accessibility Statement

The method, procedures, experimental code, and analysis scripts for each experiment were preregistered before data collection. The preregistration, code, data, and a working demo of each experiment (that does not collect data) are all available under each study component at https://osf.io/zwcjm/?view_only=f722c5c3d0d64587a6b160cc43cbe9c6.

## Additional File

The additional file for this article can be found as follows:

10.5334/joc.352.s1 Supplement Material. Post Trial Questions (exact wording).
