# Supplementary material for: Are Familiar Objects More Likely to Be Noticed in an Inattentional Blindness Task?
Source: J Cogn. 2024 Feb 22;7(1):28. doi: 10.5334/joc.352 (PMC10885827; doi:10.5334/joc.352)
Supplement: Supplement Material. — Post Trial Questions (exact wording). [file joc-7-1-352-s1.pdf]

Post Trial Questions (exact wording)

Immediately after reporting which arm of the cross was longer, participants were asked the following questions:

- "Did you notice an extra object on that trial?" [Yes | No]
  - If yes: "Describe what you saw" [open-ended text box response]
- "What was the extra object? If you didn't see one or don't know, just guess." [select from displayed versions of the possible unexpected objects for that study, with their position randomized for each participant]
- (For Experiments 3-4 only) "How frequently do you see this logo in your daily life?" [rarely or never | monthly | weekly | daily]

Following these questions, participants completed an additional "divided attention" trial that was identical to the critical trial except that the position of the cross was again determined randomly. After reporting which line was longer they answered the same questions as they had for the critical trial. Finally, participants provided demographic information and reported any playback issues through the following questions:

- "Did the animation play smoothly, with no obvious lagging or freezing?" [Yes | No]
  - If no: "Please describe the playback issues you experienced." [open-ended text box response]
- "What is your age?" [Under 18 | 18-24 | 25-49 | 50-80 | over 80]
- "Please provide the country in which you currently live." [drop-down menu list of countries]
- "Which best describes your vision?" ["My vision is normal. I don't need glasses or contacts" | "I need glasses or contacts, and I wore them during the experiment." | "I need glasses or contacts, but I wasn't wearing them during the experiment."] "Which best describes your color vision?" ["My color vision is normal" | "I am red-green colorblind" | "I am blue-yellow colorblind" | "I have some other issue with my color vision."]
- [Display of Ishihara plate 9 with a correct answer of 74] "Please select the number you see." [5 | 21 | 74 | 112 | "I don't see a number"]

- “Have you performed a similar task before, where you were asked to judge which arm of a cross was longer, and something unexpected appeared? If you have, please briefly describe it in the text box. Your answer will not affect your compensation for completing this study.” [Yes | No] [text box for “yes” response]
